# Combined effects of cigarette smoking, DNA methyltransferase 3B genetic polymorphism, and DNA damage on lung cancer

**DOI:** 10.1186/s12885-021-08800-w

**Published:** 2021-09-29

**Authors:** Chia-Chen Huang, Chung-Yu Lai, Chin-Hung Tsai, Jiun-Yao Wang, Ruey-Hong Wong

**Affiliations:** 1 Department of Public Health, Chung Shan Medical University, No. 110 Chien-Kuo N Rd., Sec. 1, Taichung, Taiwan 40242; 2 Institute of Medicine, Chung Shan Medical University, Taichung, Taiwan; 3 Department of Surgery, Cheng-Ching General Hospital, Taichung, Taiwan; 4 Center for General Education, Chung Shan Medical University, Taichung, Taiwan; 5 Division of Pulmonary and Critical Care Medicine, Department of Internal Medicine, Tungs’ Taichung MetroHarbor Hospital, Taichung, Taiwan; 6 Department of Family Medicine, Cheng Ching General Hospital, Taichung, Taiwan; 7 Department of Occupational Medicine, Chung Shan Medical University Hospital, Taichung, Taiwan

**Keywords:** Smoking, DNA damage, Green tea consumption, Lung cancer, *DNMT3B* genotype

## Abstract

**Background:**

Smoking increases DNA methylation and DNA damage, and DNA damage acts as a vital cause of tumor development. The DNA methyltransferase 3B (DNMT3B) enhances promoter activity and methylation of tumor suppressor genes. Tea polyphenols may inhibit DNMT activity. We designed a case-control study to evaluate the combined effects of smoking, green tea consumption, *DNMT3B* − 149 polymorphism, and DNA damage on lung cancer occurrence.

**Methods:**

Questionnaires were administered to obtain demographic characteristics, life styles, and family histories of lung cancer from 190 primary lung cancer cases and 380 healthy controls. Genotypes and cellular DNA damage were determined by polymerase chain reaction and comet assay, respectively.

**Results:**

The mean DNA tail moment for lung cancer cases was significantly higher than that for healthy controls. Compared to nonsmokers carrying the *DNMT3B* − 149 CT genotype, smokers carrying the TT genotype had a greater lung cancer risk (odds ratio [OR]: 2.83, 95% confidence interval [CI]: 1.62–4.93). DNA damage levels were divided by the tertile of the healthy controls’ values. Compared to nonsmokers with low DNA damage, smokers with moderate DNA damage (OR: 2.37, 95% CI: 1.54–3.63) and smokers with high DNA damage (OR: 3.97, 95% CI: 2.63–5.98) had elevated lung cancer risks. Interaction between smoking and DNA damage significantly affected lung cancer risk.

**Conclusions:**

Our study suggested that the *DNMT3B* − 149 TT genotype, which has higher promoter activity, can increase the lung cancer risk elicited by smoking, and DNA damage may further promote smoking related lung cancer development.

## Background

Lung cancer is the major cause of death from cancer around the world [1, 2]. A strong correlation of lung cancer with cigarette smoking has been established [2]. It has been suggested that the cessation of cigarette smoking reduces the risk of lung cancer development [2]. Importantly, evidence shows that smoking can increase DNA methylation and DNA damage [3, 4]. These may be the crucial mechanisms of smoking related lung cancer.

Methylation modification typically occurs in the gene promoter, especially in the CpG dinucleotide [5]. It has been shown that DNA methylation can inhibit gene expressions by directly or indirectly inhibiting the binding of protein or the transcription factor to the promoter region [5]. Previous studies have pointed out that some DNA is hypermethylated in many tumor tissues [6, 7], and such results decrease the expression of tumor suppressor genes (TSG) and regulate the cell cycle genes [8]. Abnormal methylation in a gene might also lead to chromosomal instability and sensitivity to exogenous carcinogens, thereby making the gene prone to DNA damage [9, 10]. Importantly, DNA damage has been proposed as a vital cause of cancer occurrence and development [11]. Any condition leading to high levels of DNA damage, if not repaired, may potentially result in carcinogenic effects [11].

Specifically, it has been suggested that cigarette smoking arouses DNA methylation [3, 4], causing the occurrence of several cancers, including lung cancer [3]. Smoking also induces accumulation of lots of DNA methyltransferase (DNMT) in the nucleus [12]. In the processes of DNA methylation, DNMT is a key catalyst [5–7]. The *DNMT3B* is located on chromosome 20q11.2, which contains a C-to-T transition (rs2424913) in the promoter, − 149 bp from the transcription start site [13]. This single nucleotide polymorphism (SNP) increases the promoter activity by about 30% and modulates an aberrant de-novo methylation of CpG islands in some TSG [14, 15]. So far, this polymorphism has been reported to be associated with a small number of cancers. However, correlation analyses between *DNMT3B* − 149 polymorphism and specific cancers seldom take into account the effect of environmental factors. Our previous study further revealed that the *DNMT3B* − 149 TT genotype can increase the lung cancer risk caused by smoking [16].

Another one of our studies also reported that individuals who never drink green tea have elevated lung cancer risk compared to those who drink at least one cup of green tea per day, and the effect is more pronounced in smokers [17]. Green tea has attracted considerable attention as a natural product possessing preventive effects against cancer [18]. Interestingly, an animal study showed DNA damage in the lung tissue of rats could be reduced by green tea [19]. However, the molecular mechanisms by which green tea decreases lung cancer risk are still not clear. In particular, epigallocatechin-3-gallate (EGCG), the main constituent of green tea, can inhibit DNMT activity and reactivate methylation-silenced genes [20, 21]. Moreover, DNA repair capacity in individuals is an important determinant of cancer susceptibility [11, 22]. Accumulated DNA damage causes gene instability or mutation, if the DNA damage cannot be repaired [11, 22]. However, the reverse relationship between green tea drinking and DNA damage has not been fully investigated. It is also unknown whether *DNMT3B* genotypes can modify the above relationship.

In the present study, we are interested in evaluating whether smoking, green tea consumption, and *DNMT3B* − 149 genotypes are related to the level of DNA damage in individuals. We further tested the interactions of smoking, green tea consumption, *DNMT3B* − 149 genotypes, and individual DNA damage level in the occurrence of lung cancer.

## Methods

### Case ascertainment

The design and final reports of this study complied with the Helsinki declaration and were approved by the institutional review board of the participating institutions (Chung Shan Medical University: 1031229, Taichung Cheng Ching Hospital: HP150043, Taichung Tungs’ Taichung MetroHarbor Hospital: 104072). Informed consent statements were obtained from all participants.

From August 2004 to October 2011, a total of 271 lung cancer (International Classification of Diseases, 10th revision; ICD code C33-C34) patients were recruited from participating institutions in central Taiwan. These hospitals were accessible to patients from all socioeconomic classes. Eligible cases were 20 years of age or older. All patients underwent a series of examinations of pathologic stages by board-certified pathologists. Tumor types and stages were determined according to the World Health Organization classification. Ten patients were not interviewed because of severe illness, 37 patients were not incident cases, and 34 patients were too old (range = 81–92 years) or were without complete questionnaire data. None of the included patients had been exposed to radiotherapy or chemotherapy. The demographic characteristics of excluded patients were comparable with those of included patients, with the exception of age. Among the 190 patients available for matching, cancer types were categorized as follows: 108 (56.8%) patients with adenocarcinoma, 51 (26.9%) with squamous cell carcinoma, and 31 with others (including small cell carcinoma, neuroendocrine carcinoma, mixed cell carcinoma, and unspecific malignant cell). Moreover, 132 (69.5%) patients had an onset age of older than 60 years old, 38 (20.0%) patients had an onset age of between 50 and 59 years, and 20 (10.5%) patients had an onset age of younger than 50 years old.

### Selection of controls

In this study, two controls were individually matched with each lung cancer case by age (initially ±1 year, relaxed to ±5 years) and gender. There was no familial relationship among and between cases and controls. They were also selected from the same geographic areas as the lung cancer cases. During the same period of case recruitment, 380 controls were selected randomly from participants with no history of cancer or pulmonary diseases at the time of diagnosis, including tuberculosis, pneumonia, bronchiectasis, pneumoconiosis, pulmonary alveolar pneumonopathy, chronic obstructive pulmonary disease, and asthma. They admitted to the same hospitals for physical check-ups. The primary reason why our potential controls refused to participate in the study was that most subjects were unwilling to take the time for an interview.

### Epidemiological information

Epidemiological information was collected from study participants through in-person interviews using a standardized questionnaire including demographic and lifestyle items. Subjects’ cumulative smoking dose was calculated by pack-years, defined as the number of packs of cigarettes smoked daily multiplied by the active smoking years. The same tea leaf can be brewed many times and tea is served in small cups (30–50 ml). A standard cup of tea was defined as 100–120 ml in this study. The period of exposure was assessed from birth to the day when lung cancer was first diagnosed for cases or when the interview was performed for controls. The frequency of green tea consumption was categorized as every day (more than one cup per day), three to four cups per week, one to two cups per week, one to two cups per month, and seldom. The number of cups consumed was assessed from five possible answers (for those who drank tea every day): less than one cup a day, one to two cups a day, three to four cups a day, five to nine cups a day, and ten or more cups a day. The evaluation of green tea consumption was based on a previous study [23], in which Spearman’s correlation between consumption measured by two questionnaires administered six months apart was 0.66, and the correlation between the amount of green tea consumed according to the questionnaire and the amounts consumed according to the three day in one-year food records showed the same results. Moreover, intake of fruits and vegetables was measured as the average number of standardized servings per week of fruits and vegetables over the last 3 years. For cooking exposure, participants were asked how often they used various cooking methods, including stir-frying. Family history of lung cancer was defined as lung cancer in first-degree relatives of the test participant.

### Genotyping analysis of *DNMT3B*

Venous blood from all participants was collected in heparin tubes, and prepared into plasma, buffy coat and red blood cells. Buffy coat was used to extract genomic DNA by using a Genomic DNA isolation kit (Qiagen Inc., Hilden, Germany).

Polymerase chain reaction (PCR)-restriction fragment length polymorphism was used to distinguish the variation of rs2424913 in *DNMT3B*. Primers used for the amplification were 5′-TGC TGT GAC AGG CAG AGC AG-3′ and 5′-GGT AGC CGG GAA CTC CAC GG-3′. The PCR final volume was 50 μl containing DNA (0.5 μl), PCR buffer with 200 ng of primers, 1.5 mM MgCl_2_, 0.2 mM dNTPs, 50 mM KCl, 10 mM Tris-HCl (pH 8.3), and 0.1% bovine serum albumin. Amplification conditions were the initial denaturation at 95 °C for 5 min, under 35 cycles of amplification: denaturation at 95 °C for 30 s, annealing at 63 °C for 90 s, elongation at 72 °C for 25 s, and extension at 72 °C for 40 s. The PCR products were digested with *Bfa*I at 16 h at 37 °C. Homozygous CC individuals had product fragments of 208, 126, and 46 bp, while homozygous TT individuals had product fragments of 162, 126, and 46 bp, and heterozygous CT individuals had all four fragments.

### Comet assay

The comet assay was conducted under alkali conditions according to the procedure of Singh et al. [24]. The 10 μl of whole blood was suspended in 1.5% low-melting point agarose and sandwiched between a layer of 0.6% normal-melting agarose and a top layer of 1.5% low-melting point agarose on fully frosted slides. The slides were lysed in lysis solution (1% sodium sarconisate, 2.5 M NaCl, 100 mM Na_2_EDTA, 10 mM Tris-HCl, 1% Triton X-100, and 10% DMSO) for 1 h at 4 °C. Slides were placed in buffer (0.3 mol/L NaOH, 1 mmol/L Na_2_EDTA, pH 13) for 15 min. Next, the slides were washed three times for 5 min with PBS, moved to an electrophoresis tank, and then stained with 10% ethidium bromide. For each participant, 100 randomly captured comets from slides (25 cells on each of four comet slides) were examined at × 400 magnification using an epifluorescence microscope connected through a black and white camera to an image analysis system (Comet Assay II; Perceptive Instruments Ltd., Haverhill, Suffolk, UK). A computerized image analysis system acquired images, integrated intensity profiles, estimated the comet cell components, and evaluated the range of derived variables for each cell. Undamaged cells have an intact nucleus and damaged cells have the appearance of a comet. Tail moment was calculated as the product of the tail length and the DNA fraction in the comet tail to quantify DNA damage. All slides were counted by a reader who was blind to the status of participants.

### Statistical analysis

Initially, this study estimated four sample sizes. The same type I (α) error of 0.05, type II (β) error of 0.2, and odds ratio (OR) of 1.8 were given. According to the prevalence of smoking status, green tea consumption, *DNMT3B* − 149 T allele in a previous study [17], and DNA damage levels would be divided into three groups by the tertile of the healthy controls’ values, we assumed that the corresponding exposure prevalence in the control group to the above factors was 31, 66, 95, and 33%, respectively. Further, the minimum sample size required for the case group for each factor was 145, 175, 983, and 141, respectively. In general, increase in sample size for both case and control groups leads to a greater statistical power to detect a significant difference. Additional controls per case also need to be considered for stratified analyses, in which each case together with its matched controls constitutes a distinct stratum. Considering limited availability of resources and efficiency, this study decided to collect at least 175 cases, and set the control-case ratio of 2.

All data were analyzed using SAS 9.6 software (SAS Institute, Cary, NC, USA). The normal distribution of the continuous variables was checked by the Kolmogorov-Smirnov test. Subsequently, comparisons between the case and control groups were made using a Student’s *t* test for the age variable and a χ^2^-test or Fisher’s exact test for discrete variables. Hardy-Weinberg equilibrium was performed to test *DNMT3B* − 149 genotypes for the goodness of fit χ^2^-test. Because of the positively skewed distribution of the DNA damage level, the Mann-Whitney U test and Kruskal-Wallis test were used to test the level differences for each variable. Mean and median values of the DNA damage level were also presented. Backward stepwise log-linear regression analysis was performed to reduce the full model to a more parsimonious final model, and adjusted OR and a 95% confidence interval (95% CI) were obtained for each variable. Further, likelihood ratio χ^2^-tests were utilized to test the interaction between two variables with respect to the risk of lung cancer. All tests were two-tailed, and all *p* values were considered to indicate statistical significance.

## Results

### Participant characteristics

In total, 570 participants were recruited in this study (60.5% for males and 39.5% for females), and the characteristics of this study participants are summarized in Table 1. At recruitment, the mean age was 65.5 years for lung cancer patients and 64.4 years for healthy controls (range = 29–93 years). As expected, the lung cancer patients included more smokers when compared with healthy controls (53.7% vs 31.1%, OR: 2.20, 95% CI: 1.84–2.63), and the proportions of those with more than 40 pack-years of smoking for lung cancer patients and healthy controls were 34.2 and 15.8% (OR: 2.46, 95% CI: 2.43–2.50), respectively. More nondrinkers of green tea presented as lung cancer patients compared with healthy controls (76.8% vs 65.8%, OR: 1.71, 95% CI: 1.68–1.73). Further, 16.6% of healthy controls consumed green tea for more than 10 years, but only 11.1% of lung cancer patients drank green tea for more than 10 years. A difference in fruits and vegetables intake of less than 14 servings per week was observed between the lung cancer cases and controls (25.3% vs 36.6%, OR: 0.80, 95% CI: 0.78–0.81). Compared with controls, exposure to cooking fumes and family history of lung cancer were both observed at significantly higher frequencies in lung cancer patients. Moreover, more lung cancer cases than controls were *DNMT3B* − 149 TT genotype carriers (96.3% vs 90.8%, OR: 1.62, 95% CI: 1.35–1.94).

**Table 1:** The distributions of specific characteristics by cases and controls status

Variables	Cases	Controls	
	***N*** **= 190 (%)**	***N*** **= 380 (%)**	**OR (95% CI)**^**a**^
Gender			
Male	115 (60.5%)	230 (60.5%)	1.00 (0.84–1.20)
Female	75 (39.5%)	150 (39.5%)	Ref.
Age (years; mean ± SD)	65.5 ± 11.9	64.4 ± 11.8	
≥ 60	132 (69.5%)	252 (66.3%)	1.02 (1.01–1.04)^**^
51–59	38 (20.0%)	88 (23.2%)	0.93 (0.78–1.11)
≤ 50	20 (10.5%)	40 (10.5%)	Ref.
Smoking status			
Current or ever smokers	102 (53.7%)	118 (31.1%)	2.20 (1.84–2.63)^***^
Nonsmokers	88 (46.3%)	262 (68.9%)	Ref.
Pack-years smoked			
≥ 40	65 (34.2%)	60 (15.8%)	2.46 (2.43–2.50)^***^
1–39	37 (19.5%)	58 (15.3%)	1.90 (1.59–2.28)^***^
0	88 (46.3%)	262 (68.9%)	Ref.
Green tea consumption (cup/day)			
0	146 (76.8%)	250 (65.8%)	1.71 (1.68–1.73)^***^
< 1	29 (15.3%)	54 (14.2%)	1.65 (1.38–1.98)^**^
≥ 1	15 (7.9%)	76 (20.0%)	Ref.
Green tea consumption (years)			
0	146 (76.8%)	250 (65.8%)	1.32 (1.30–1.34)^*^
≤ 10	23 (12.1%)	67 (17.6%)	1.02 (0.86–1.23)
> 10	21 (11.1%)	63 (16.6%)	Ref.
Fruits and vegetables intake			
≤ 14	48 (25.3%)	139 (36.6%)	0.80 (0.78–0.81)^*^
15–20	51 (26.8%)	69 (18.2%)	1.18 (0.99–1.41)
≥ 21	91 (47.9%)	172 (45.2%)	Ref.
Exposure to cooking fumes (hours/week)			
≥ 3	17 (8.9%)	16 (4.2%)	1.64 (1.61–1.66)^***^
1–3	19 (10.0%)	15 (4.0%)	1.85 (1.55–2.21)^***^
< 1	154 (81.1%)	349 (91.8%)	Ref.
Family history of lung cancer			
Yes	15 (7.9%)	6 (1.6%)	2.38 (1.99–2.84)^***^
No	175 (92.1%)	374 (98.4%)	Ref.
*DNMT3B* − 149 genotypes			
TT	183 (96.3%)	345 (90.8%)	1.62 (1.35–1.94)^*^
CT	7 (3.7%)	35 (9.2%)	Ref.
CC	0 (0.0%)	0 (0.0%)	
T allele	373 (98.2%)	725 (95.4%)	1.63 (1.21–2.18)^*^
C allele	7 (1.8%)	35 (4.6%)	Ref.
Histological type			
Adenocarcinoma	108 (56.8%)		
Squamous cell carcinoma	51 (26.9%)		
Others^b^	31 (16.3%)		

Abbreviation: *N* number; *OR* odds ratio; *CI* confidence interval; *Ref*. reference

^a^Data were matched by age and gender

^b^Others included small cell carcinoma, large cell carcinoma, mixed cell carcinoma, and unspecific malignant cell

^*^0.01 < *p* < 0.05, ^**^0.001 < *p* < 0.01, ^***^p < 0.001

### DNA tail moment of lung cancer patients and controls

Table 2 shows the mean DNA tail moment of each peripheral blood cell with stratification of specific characteristics in lung cancer patients and controls. The DNA tail moment in lung cancer patients was significantly higher than that of controls (mean: 1.38 [median 1.17] vs 1.00 [0.98] μm, *p* < 0.001, Mann-Whitney U test). The tail moment was not associated with various factors in lung cancer cases. We did not observe the correlation between various factors and DNA tail moment in healthy controls, with the exception of smoking habits. In the control group, smokers had lower DNA tail moments than nonsmokers (median: 0.89 vs 1.00 μm, *p* < 0.01). Similarly, healthy controls with cumulative smoking of more than 40 pack-years and 1–39 pack-years also had significantly lower DNA tail moments than did nonsmokers (0.90, 0.89 vs 1.00 μm, *p* < 0.01, Kruskal-Wallis test). Further, we divided the DNA damage levels into high, moderate, and low groups by the tertile of the healthy controls’ values (Table 3). Compared to those with low levels of DNA damage, subjects with high levels of DNA damage had a greater OR of 1.70 (95% CI: 1.34–2.15) for lung cancer.

**Table 2:** The DNA tail moment per cell with stratification of specific characteristics in lung cancer cases and controls

Variables	Cases		Controls	
	N	Mean (median) ± SD	N	Mean (median) ± SD
All	190	1.38 (1.17) ± 1.15	380	1.00 (0.98) ± 0.33^b*^
Gender				
Male	115	1.44 (1.18) ± 0.67	230	0.99 (0.96) ± 0.33
Female	75	1.28 (1.14) ± 0.85	150	1.03 (0.99) ± 0.34
Age				
≥ 60	132	1.38 (1.15) ± 1.27	252	1.02 (0.99) ± 0.33
51–59	38	1.44 (1.27) ± 0.90	88	0.94 (0.96) ± 0.32
≤ 50	20	1.23 (1.15) ± 0.70	40	1.02 (0.99) ± 0.36
Smoking status				
Current or ever smokers	102	1.32 (1.15) ± 0.71	118	0.91 (0.89) ± 0.32^c*^
Nonsmokers	88	1.44 (1.19) ± 1.51	262	1.04 (1.00) ± 0.33
Pack-years smoked				
≥ 40	65	1.31 (1.19) ± 0.62	60	0.88 (0.90) ± 0.32^c*^
1–39	37	1.34 (1.14) ± 0.85	58	0.94 (0.89) ± 0.32
0	88	1.44 (1.19) ± 1.51	262	1.04 (1.00) ± 0.33
Green tea consumption (cup/day)				
0	146	1.29 (1.17) ± 0.74	250	1.02 (0.98) ± 0.34
< 1	29	1.34 (1.15) ± 0.67	54	0.98 (0.97) ± 0.34
≥ 1	15	2.31 (1.32) ± 3.22	76	0.98 (0.98) ± 0.31
Green tea consumption (years)				
0	146	1.29 (1.17) ± 0.74	250	1.02 (0.98) ± 0.34
≤ 10	23	1.15 (1.14) ± 0.51	67	0.94 (0.96) ± 0.31
> 10	21	2.24 (1.55) ± 2.73	63	1.02 (1.02) ± 0.32
Fruits and vegetables intake				
≤ 14	48	1.50 (1.26) ± 0.83	139	0.95 (0.95) ± 0.35
15–20	51	1.25 (1.21) ± 0.60	69	1.01 (0.95) ± 0.33
≥ 21	91	1.39 (1.09) ± 1.49	172	1.04 (1.01) ± 0.32
Exposure to cooking fumes (hours/week)				
≥ 3	17	1.35 (1.14) ± 0.92	16	1.11 (1.10) ± 0.36
1–3	19	1.14 (1.09) ± 0.57	15	0.96 (0.99) ± 0.26
< 1	154	1.41 (1.18) ± 1.23	349	1.00 (0.97) ± 0.33
Family history of lung cancer				
Yes	15	1.12 (1.08) ± 0.67	6	0.98 (0.92) ± 0.16
No	175	1.40 (1.18) ± 1.18	374	1.00 (0.98) ± 0.34
*DNMT3B* − 149 genotypes				
TT	183	1.32 (1.17) ± 0.75	345	1.00 (0.98) ± 0.33
CT	7	2.99 (1.19) ± 4.63	35	1.03 (1.06) ± 0.33
Histological type				
Adenocarcinoma	108	1.42 (1.18) ± 1.39		
Squamous cell carcinoma	51	1.39 (1.19) ± 0.75		
Others^a^	31	1.22 (0.97) ± 0.70		

Abbreviation: *N* number; *SD* standard deviation

^a^Others included small cell carcinoma, large cell carcinoma, mixed cell carcinoma, and unspecific malignant cell

^b^Data was calculated by Mann-Whitney U test to examine the difference of DNA tail moment between the case and control groups

^c^Data were calculated by Mann-Whitney U test and Kruskal-Wallis test to examine the differences of DNA tail moment for each variable in controls

^*^p < 0.001

**Table 3:** The DNA damage associated with lung cancer risk

Variables	Cases	Controls	
	*N* = 190 (%)	*N* = 380 (%)	**OR (95% CI)**
DNA damage level			
High	106 (55.8%)	129 (33.9%)	1.70 (1.34–2.15)^*^
Moderate	39 (20.5%)	125 (32.9%)	1.01 (0.77–1.32)
Low	45 (23.7%)	126 (33.2%)	Ref.

Abbreviation: *N* number; *OR* odds ratio; *CI* confidence interval; *Ref*. reference

Data were matched by age and gender, calculated by backward stepwise log-linear regression, adjusted for pack-years smoked, green tea consumption, and exposure to cooking fumes

^*^p < 0.001

### Joint effects of smoking status with *DNMT3B* − 149 genotypes and DNA damage level on lung cancer risk

Subsequently, we respectively analyzed the joint effects of smoking status with *DNMT3B* − 149 genotypes and DNA damage level on lung cancer risk (Table 4). Compared with nonsmokers carrying the *DNMT3B* − 149 CT genotype, smokers carrying the *DNMT3B* − 149 TT genotype had a higher lung cancer risk (OR: 2.83, 95% CI: 1.62–4.93). A borderline significant interaction between smoking status and *DNMT3B* − 149 genotypes on lung cancer risk was observed (*p* = 0.06). Smoking status was replaced by cumulative smoking dose in subsequent analysis. A borderline significant interaction between cumulative smoking dose and *DNMT3B* − 149 genotypes on lung cancer risk was still maintained (*p* = 0.07). Similarly, nonsmokers with a low level of DNA damage were selected as the reference group. Smokers with a moderate level of DNA damage (OR: 2.37, 95% CI: 1.54–3.63) and a high level of DNA damage (OR: 3.97, 95% CI: 2.63–5.98) had significantly increased risks of lung cancer. A significant interaction between smoking status and DNA damage level on lung cancer risk was further observed (*p* < 0.01). Moreover, the test showed interaction between cumulative smoking and DNA damage level also significantly affected lung cancer risk (*p* = 0.01).

**Table 4:** The joint effects of smoking status with *DNMT3B* − 149 genotypes and DNA damage level on lung cancer risk

Variables	Smoking status				Pack-years of smoked					
	Nonsmokers		Current and ever smokers		0		1–39		≥ 40	
	**CA/CN**	**OR (95% CI)**^a^	**CA/CN**	**OR (95% CI)**^a^	**CA/CN**	**OR (95% CI)**^a^	**CA/CN**	**OR (95% CI)**^a^	**CA/CN**	**OR (95% CI)**^a^
*DNMT3B* − 149 genotypes										
TT	83/242	1.09 (0.64–1.86)	100/103	2.83 (1.62–4.93)^***^	83/242	1.09 (0.64–1.86)	37/52	2.39 (1.33–4.29)^**^	63/51	3.20 (1.80–5.67)^***^
CT	5/20	1.00 (Ref.)	2/15	1.08 (0.43–2.75)	5/20	1.00 (Ref.)	0/6	–	2/9	1.46 (0.56–3.82)
Test for interaction		χ^2^ = 3.64 (1 df); *p* = 0.06			χ^2^ = 5.43 (2 df); *p* = 0.07					
DNA damage level										
High	49/100	1.21 (0.89–1.66)	57/29	3.97 (2.63–5.98)^***^	49/100	1.21 (0.89–1.66)	19/17	3.03 (1.86–4.96)^***^	38/28	4.90 (3.05–7.88)^***^
Moderate	15/90	0.66 (0.45–0.98)^*^	24/35	2.37 (1.54–3.63)^***^	15/90	0.66 (0.45–0.98)^*^	9/15	2.10 (1.21–3.65)^**^	15/20	2.54 (1.56–4.13)^***^
Low	24/72	1.00 (Ref.)	21/54	1.56 (1.03–2.35)^*^	24/72	1.00 (Ref.)	9/26	1.42 (0.86–2.36)	12/12	1.68 (1.04–2.71)^*^
Test for interaction		χ^2^ = 11.80 (2 df); *p* < 0.01			χ^2^ = 12.42 (4 df); *p* = 0.01					

Abbreviation: *CA/CN* case numbers/control numbers; *OR* odds ratio; *CI* confidence interval; *Ref.* reference; *df* degree of freedom

^a^Data were matched by age and gender, calculated by backward stepwise log-linear regression, and adjusted for green tea consumption, fruits and vegetables intake, and exposure to cooking fumes

^*^0.01 < *p* < 0.05, ^**^0.001 < *p* < 0.01, ^***^p < 0.001

### Joint effects of green tea consumption, *DNMT3B* − 149 genotypes and DNA damage level on lung cancer risk

Last, we analyzed the joint effects of green tea consumption, *DNMT3B* − 149 genotypes and DNA damage level on lung cancer risk (Table 5). However, no significant association with lung cancer risk was found for any combinations of different green tea drinking status and drinking durations with *DNMT3B* − 149 genotypes. When drinkers with a low level of DNA damage were selected as the reference group, non-drinkers with a low level of DNA damage had a 1.80-fold (95% CI: 1.15–2.82) greater risk of lung cancer. Among those with a high level of DNA damage, green tea drinkers (OR: 2.26, 95% CI: 1.41–3.63) and non-drinkers (OR: 2.75, 95% CI: 1.78–4.25) had significantly increased risks of lung cancer. Drinking status was further stratified by consumption duration, and those with a low level of DNA damage and >  10 years of green tea consumption were selected as the reference group. Non-drinkers with a low level of DNA damage had a 2.17-fold (95% CI: 1.28–3.68) greater risk of lung cancer. Among the subjects expressing a high level of DNA damage, those who consumed green tea > 10 years (OR: 2.64, 95% CI: 1.82–3.83), ≤ 10 years (OR: 2.82, 95% CI: 1.97–4.03), and non-drinkers (OR: 3.31, 95% CI: 2.55–4.30) had significantly increased risks of lung cancer. However, no significant interaction between green tea consumption and DNA damage level on lung cancer risk was found.

**Table 5:** The joint effects of green tea consumption with *DNMT3B* − 149 genotypes and DNA damage level on lung cancer risk

Variables	Drinking status				Drinking duration in years					
	Drinkers		Non-drinkers		> 10		≤ 10		0	
	**CA/CN**	**OR (95% CI)**^a^	**CA/CN**	**OR (95% CI)**^a^	**CA/CN**	**OR (95% CI)**^a^	**CA/CN**	**OR (95% CI)**^a^	**CA/CN**	**OR (95% CI)**^a^
*DNMT3B* -149 genotypes										
TT	42/121	1.47 (0.65–3.31)	141/224	2.15 (0.96–4.79)	19/58	0.94 (0.38–2.33)	23/63	1.12 (0.45–2.77)	141/224	1.50 (0.62–3.62)
CT	2/9	1.00 (Ref.)	5/26	1.22 (0.48–3.12)	2/5	1.00 (Ref.)	0/4	–	5/26	0.86 (0.32–2.33)
Test for interaction		χ^2^ = 0.13 (1 df); *p* = 0.72			χ^2^ = 3.05 (2 df); *p* = 0.22					
DNA damage level										
High	26/40	2.26 (1.41–3.63)^**^	80/89	2.75 (1.78–4.25)^**^	13/20	2.64 (1.82–3.83)^**^	13/20	2.82 (1.97–4.03)^**^	80/89	3.31 (2.55–4.30)^**^
Moderate	10/42	1.16 (0.68–1.96)	29/83	1.77 (1.12–2.80)^*^	5/23	1.29 (0.82–2.05)	5/19	1.51 (0.94–2.42)	29/83	2.13 (1.38–3.29)^*^
Low	8/48	1.00 (Ref.)	37/78	1.80 (1.15–2.82)^*^	3/20	1.00 (Ref.)	5/28	1.39 (0.88–2.17)	37/78	2.17 (1.28–3.68)^*^
Test for interaction		χ^2^ = 2.25 (2 df); *p* = 0.32			χ^2^ = 2.61 (4 df); *p* = 0.63					

Abbreviation: CA/CN case numbers/control numbers; *OR* odds ratio; *CI* confidence interval; *Ref.* reference; *df* degree of freedom

^a^Data were matched by age and gender, calculated by backward stepwise log-linear regression, and adjusted for pack-years of smoked, and exposure to cooking fumes

^*^0.01 < *p* < 0.05, ^***^p < 0.001

## Discussion

As in our previous studies [16, 17], independent effects of smoking, green tea consumption, and *DNMT3B* − 149 genotypes on the development of lung cancer were observed. In the current study, DNA damage level for lung cancer cases was significantly higher than that for healthy controls. Significant effects of the interaction between smoking and DNA damage level on lung cancer risk were further revealed.

The comet assay is widely used in studies on genotoxicity testing, but rarely used in cancer epidemiological research [25]. Interestingly, the current epidemiological study showed that the median DNA tail moment for lung cancer cases was significantly higher than that of healthy controls. In our study, none of all included lung cancer patients had been exposed to radiotherapy or chemotherapy and all healthy controls had no history of cancer or pulmonary diseases before collecting blood samples. This was consistent with an early comparative study [26], in which the mean DNA tail moment of peripheral lymphocytes that had not been exposed to radiation in lung cancer patients was significantly higher than that in controls. Further, evidence notes that smoking increases DNA methylation and DNA damage [3, 4]. Abnormal methylation in genes may also lead to chromosomal instability and sensitivity to exogenous carcinogens, thereby making genes prone to DNA damage [9, 10]. These may be the crucial mechanisms of smoking related lung cancer. However, DNA damage was not associated with various factors in our cases with lung cancer, and cancer-free smokers had a lower level of DNA damage than did nonsmokers. Such a result could shed light on the roles of other factors that we have not explored in this study, such as metabolism of cigarette smoke components and the repairing of DNA damage. Lung cancer patients might also present with DNA damage in blood cells due to their poor antioxidant defense state and greater oxidative stress in the body [27]. The observed DNA damage can be regarded as the overall effect of these unexplored factors, which showed significant effects on lung cancer risk in this study. It is worth mentioning that some smokers may partially compensate for nicotine use [28, 29]. These smokers may adapt their smoking behavior to obtain a certain smoke (nicotine) dose for each cigarette. When compensating for low nicotine yields by smoking cigarettes more intensively, smokers also take in larger amounts of carcinogens from each cigarette, causing even greater health hazards [28, 29]. However, it is questionable whether a single measurement can adequately represent the exposure of participants to carcinogens. Another explanation for the current observations could be the possibility of recall bias in self-reported smoking data, thereby causing exposure misclassification. On the contrary, our cancer-free nonsmokers showed detectable cellular DNA damage. It is possible that, with none or very low exposures to cigarette smoke, the biology that results in DNA damage is driven by endogenous carcinogens. This could also reflect background levels due to other kinds of exposure. However, no information was available on potential exposure to tobacco smoke or products, such as nonsmokers living with smokers or working with smokers or occupational exposure to smoke or automotive exhaust/diesel fumes.

It is reasonable to assert that the *DNMT3B* polymorphism is associated with cancer development by increasing the promoter activity of *DNMT3B* and modulating an aberrant de novo methylation of CpG islands in some TSG [14, 15]. However, the effect of *DNMT3B* − 149 C to T on *DNMT3B* expression is still unclear. As expected, the independent effect of *DNMT3B* − 149 genotypes on the development of lung cancer in Taiwanese cases was observed. A study conducted in a non-Hispanic Caucasian population also showed that *DNMT3B* − 149 T allele was associated with increased lung cancer risk [13]. However, another study did not find that this allele was associated with lung cancer risk among a Chinese population [30]. The inconsistent findings might be due to different ethnic populations and gene expressions at distinct tumor stages. Variations in genetic background and/or environmental exposure can lead to divergent results in the development of lung cancer among distinct ethnicities. Selection bias might also exist in the aforementioned studies.

The present study found that the combined effect between smoking and *DNMT3B* − 149 genotypes on lung cancer risk is significant, although the interaction only reached marginal statistical significance. The interaction of smoking and DNA damage level of individuals also significantly affected lung cancer risk, according to the present study. Such epidemiological evidence suggests that smoking elevates lung cancer risk by increasing long-term carcinogen exposure, and simultaneously increases DNA methylation levels, providing a further opportunity to induce cancer. However, as mentioned above, DNA damage was not shown to be associated with various factors in the lung cancer group in this study, and the observed DNA damage can be regarded as the overall effect of unexplored factors. Environmental exposure to exogenous substances may lead to covalent bonding to DNA, which in turn may result in chromosomal variation; this may be the crucial step in chemical carcinogenesis [11]. Individual DNA repair capacity is a crucial determinant of cancer susceptibility [11, 22]. Accumulated DNA damage may lead to genetic mutation or genetic instability, if the DNA damage caused by carcinogens is not repaired [11, 22]. Based on this speculation, it is reasonable to assume that individuals exposed to smoking and other risk factors simultaneously will be more likely to develop lung cancer. Taken together, the *DNMT3B* − 149 TT genotype, which has higher promoter activity, could increase the lung cancer risk elicited by cigarette smoking, and greater DNA damage might further promote smoking related lung cancer development. Further studies are needed to clarify the above speculation.

Tea polyphenols may prevent mutagenicity and genotoxicity, inhibit tumor initiation, promotion, and cell proliferation, regulate detoxifying enzymes, and trap activated metabolites of carcinogens [18, 31]. Moreover, EGCG of tea polyphenols inhibit DNMT activity and thus reduce tumors in different tissues or cancer cells [32, 33]. In the present epidemiological study, an independent effect of green tea consumption on the development of lung cancer was observed. The significant combined effect of green tea consumption and DNA damage level on lung cancer risk was further revealed, although the interaction was not significant. However, the present study could not detect a significant combined effect between green tea consumption and *DNMT3B* − 149 genotypes on lung cancer risk. Previously, an animal study showed DNA damage in the lung tissue of rats could be prevented by green tea [19]. Our observations might point to the clue that tea polyphenols emerge as putative preventives and coadjuvants in the treatment of lung cancer related to DNA damage. Such speculation needs to be confirmed, and may be a less relevant mechanism in lung cancer development. In addition, misclassification may also occur, because information about green tea drinking has been obtained from questionnaires in most epidemiological studies, including our study.

In the current study, exposure to cooking fumes was associated with lung cancer risk. It is well-known that oil fumes from stir fry cooking, along with concentration of oil fumes due to poor ventilation, are associated with lung cancer [34]. Moreover, lung cancer cases have a higher proportion with a family history of lung cancer than do the controls in our study. This result indicated that familiar risk of lung cancer could be due to genetic factors or common environmental factors. Many studies suggest that the intake of fruits and vegetables is beneficial for the prevention of lung cancer, but the observed association between the intake of fruits and vegetables and the risk of lung cancer is controversial [35, 36]. In our study, no correlation between the intake of fruits and vegetables and lung cancer risk was observed. Moreover, those who consumed less fruits and vegetables actually had a lower risk of lung cancer. The possible reason could be the difficulty to accurately estimate the actual intake of fruits and vegetables by using a questionnaire, as most studies do.

Although the mechanism of comet formation observed in comet assay has not been fully clarified, it has been widely used to assess DNA damage in cells [24, 37]. However, it should be noted that DNA damage in blood cells might not be a good representative of the DNA damage of lung cells. In our study, the dispersion coefficient (0.33) of the DNA tail moment for healthy controls was close to that shown in a previous study (0.40) [38]. The *DNMT3B* − 149 T allele frequency was 95.4% in our healthy controls, which is similar that found in a Chinese report (97.8%) [39]. The frequency of *DNMT3B* − 149 genotypes also fell within Hardy-Weinberg equilibrium in the control group. These findings should confirm the credibility and results of our experimental technology. However, since the frequency of the *DNMT3B* − 149 CC genotype was small, there may have been false positive results. Additionally, the expressions of *DNMT3B* were not measured in this study. Therefore, it is necessary to increase the sample size and design more effective methods to confirm our results in the future. Our research may also have been subject to selection bias, because when healthy persons go to hospitals for physical check-ups, they may have healthier behavior. Previously, this study estimated the sample size based on given parameters, including β error of 0.2. Actually, the present study collected 190 lung cancer cases. According to smoking status, green tea consumption, *DNMT3B* − 149 genotypes, and high DNA damage level, the hazardous exposure prevalence in the control group to the above factors was 31.1, 65.8, 90.8, and 33.9%. The corresponding statistical power was 99.1, 76.8, 14.5, and 83.3%, respectively. Obviously, the statistical power of a single genotype is quite insufficient. After stratified analysis, the statistical power of risk factors for lung cancer risk was also limited due to the small sample size in this study.

## Conclusions

On the whole, this study suggested that the *DNMT3B* − 149 TT genotype, which has higher promoter activity, could increase the lung cancer risk elicited by cigarette smoking, and greater DNA damage might further promote smoking related lung cancer development.

## Data Availability

The data that support the findings of this study are available from the corresponding author upon reasonable request.

## Abbreviations

DNMT: DNA methyltransferase
DNMT3B: DNA methyltransferase 3B
TSG: Tumor suppressor genes
SNP: Single nucleotide polymorphism
EGCG: Epigallocatechin-3-gallate
PCR: Polymerase chain reaction
ANOVA: Analysis of variance
OR: Odds ratio
CI: Confidence intervals
SD: Standard deviation
GLM: General linear models
ref.: reference
df: degree of freedom
